# The Performance and Mechanism of a Mg-Al Double-Layer Oxide in Chloride ion Removal from an Aqueous Solution

**DOI:** 10.3390/nano12050846

**Published:** 2022-03-02

**Authors:** Xueqin Xu, Peng Li, Shichong Yang, Tong Zhang, Xiangke Han, Guoli Zhou, Yijun Cao, Daoguang Teng

**Affiliations:** 1 Henan Province Industrial Technology Research Institution of Resources and Materials, Zhengzhou University, Zhengzhou 450001, China; xxq18838180617@163.com; 2 School of Chemical Engineering, Zhengzhou University, Zhengzhou 450001, China; zdhglipeng@zzu.edu.cn (P.L.); yscofzzu@163.com (S.Y.); yoygat@163.com (T.Z.); xiangkehan@163.com (X.H.); teng_daoguang@zzu.edu.cn (D.T.)

**Keywords:** Mg-Al LDO, Cl^−^ removal, adsorption kinetics, electrostatic adsorption, reusability

## Abstract

The increasing threat of chloride ions (Cl^−^) has led researchers to explore efficient removal technologies. Sewage treatment with a double-layer hydroxide/oxide (LDH/LDO) is receiving increasing attention. In this work, Mg-Al LDO adsorbents were produced by the calcination of the Mg-Al LDH precursor, which was constituted by improved coprecipitation. The influence of calcination temperature, calcination time, adsorbent dosage, Cl^−^ initial concentration, contact time, and adsorption temperature on Cl^−^ elimination was investigated systematically. The experimental results showed that a better porous structure endowed the Mg-Al LDO with outstanding adsorption properties for Cl^−^. The adsorption process was well matched to the pseudo-second-order kinetics model and the Freundlich model. Under optimal conditions, more than 97% of the Cl^−^ could be eliminated. Moreover, the removal efficiency was greater than 90% even after 11 adsorption–desorption cycles. It was found that the electrostatic interaction between Cl^−^ and the positively charged Mg-Al LDO laminate, coupled with the reconstruction of the layer structure, was what dominated the Cl^−^ removal process.

## 1. Introduction

Large quantities of chloride wastewater are generated by the metallurgical and chemical industries [1,2]. Cl^−^ concentration in this wastewater is much greater than 2000 mg L^−1^, even up to 10,000 mg L^−1^ [3]. It is the high fluidity, activity, and stability of Cl^−^ that make it difficult to remove [4]. Cl^−^ can destroy the passive film to corrode the pipelines of the water circulation system when it has frequent exposure to metal [3,5]. Furthermore, most of the scale and corrosion inhibitors cannot really inhibit Cl^−^ corrosion during the process of production. At present, being dependent on the first-level water discharge requirements of the salt chemical industry, the Cl^−^ content must be kept below 300 mg L^−1^. Therefore, it is necessary and important to find a green treatment technology for Cl^−^ in the wastewater.

Great effort has been made toward alleviating the dire situation of Cl^−^ concentration in wastewater, including precipitation [3,4,6], evaporation and concentration [7], oxidation–reduction [8,9], and ion exchange [10]. However, it is worth noting that the methods described above are hard to practically apply to industrial wastewater due to prohibitive operating costs, inefficiency, limitations of treatment concentration, and a lack of suitable oxidants. By contrast, adsorption is gaining attention due to the fact that it does not harm the environment, has a straightforward process, is low in cost, and is highly efficient [11,12]. Hydrotalcite-like compounds, inorganic functional materials with layered structures, are considered to be highly promising, highly efficient adsorbents due to several factors. Their theoretical specific surface areas are large, the hydrophilicity is strong, the interlayer charge density and reactivity is high, the economic consumption is low, and efficiency is satisfactory [11].

LDHs, also known as hydrotalcites, are a class of two-dimensional anionic intercalation materials with a bimetallic hydroxide laminate and exchangeable intercalation anions [13]. They are widely applied in adsorption, catalysis [14,15,16], energy storage [17,18], biology [19], medicine release [20,21,22], heavy metal ions [23,24], and functional materials [25,26,27,28]. Their general formula can be given by (M^2+^_1−x_M^3+^_x_(OH)_2_) (A^n−^)_x/n_·mH_2_O [29]. Their laminate elements are characteristically available in variable types and various proportions; changeable interlayer anions and controllable sizes also bring more possibility in terms of application of the materials [30,31]. Anions such as NO_3_^−^ can be exchanged with CO_3_^2−^, SO_4_^2−^, and Cl^−^. In addition, LDHs that are calcined can undergo structural transformation and convert into metal oxides (LDOs) with thermal and chemical stability. When LDHs are calcined, most of the interlayer anions are eliminated and then can be incorporated during rehydration to reconstruct the LDOs into their original layered structure, which is called “the structure memory effect.” It is possible to recover the layered structure of mixed oxides when they come in contact with water [32]. The exchange between negative anions in the aqueous solution and the remaining anions in the interlayer can further improve the removal efficiency of the target ions. LDO is becoming a popularly used adsorbent in wastewater treatment [33,34].

In recent years, more research has been conducted on LDH. Most of them were focused on the impact of the component and structure, preparation and modification methods of hydrotalcite, and the application of functionalized LDH [35,36,37,38,39,40]. However, there are limited studies that focus on the absorption of Cl^−^ by LDO [41,42,43,44]. Therefore, it is crucial to provide current research and update previous findings on the removal of Cl^−^ using double-layer hydroxide/oxide materials as adsorbents.

Within this paper, an improved coprecipitation method [45] was adopted in order to fabricate the Mg-Al LDH precursor and then the adsorbent was produced by the calcination of that precursor. The structure of this adsorbent was adapted by changing the M^2+^/M^3+^ and the calcination temperature and time. The properties of the materials were studied by the systematic characterization and batch experiments. The influence of different calcination temperatures, calcination time, adsorbent doses, Cl^−^ initial concentration, contact time, and adsorption temperatures on Cl^−^ removal efficiency was explored. These findings were used to clarify the adsorption law and the adsorption mechanism of Mg-Al LDO on Cl^−^ in water. In addition, stability and reusability of LDO materials were studied.

## 2. Experiment

### 2.1. Reagents

Sodium hydroxide (NaOH) was purchased from Sinopharm Chemical Reagent Co., Ltd. (Shanghai, China). Aluminum nitrate was purchased from Shanghai Macklin Biochemical Co., Ltd., and magnesium nitrate was obtained from Tianjin Kermel Reagent Technologies Co., Ltd., Tianjin, China. All of these chemicals were of analytical purity, and they were used without further purification during the entire experiment. Magnesium nitrate (Mg (NO_3_)_2_·6H_2_O), aluminum nitrate (Al (NO_3_)_3_·9H_2_O), and sodium hydrate (NaOH) were used to prepare the LDH precursor. Sodium chloride (NaCl) was used to prepare the standard solution of chloride. Deionized water was used throughout the experiment.

### 2.2. Preparation of the Adsorbents

The adsorbents used were produced by the calcination of the Mg-Al LDH precursor, which was fabricated by improved coprecipitation implemented at room temperature [45]. The calcination temperature and the calcination time were optimized during the process (as shown in Figures S1 and S2).

When the molar ratio of M^3+^/ (M^2+^ + M^3+^) was between 0.2 and 0.4 [46], a structured LDH could be acquired. In addition, considering the standardization and rationality of the experimental design, the M^2+^:M^3+^ molar ratios of 2:1, 3:1, 4:1, 5:1, and 6:1 were chosen to fabricate the precursor. Based on the molar ratio, different masses of Mg (NO_3_)_2_·6H_2_O and Al (NO_3_)_3_·9H_2_O were mixed together to prepare 250 mL of a mixed metal nitrate solution (A). The salt solution (A) and an alkaline solution (B) (NaOH) = 3 M (250 mL) were rapidly mixed, and a white slurry was formed synchronously. The solution obtained was kept aging for 18 h at room temperature, and the supernatant was divided by filtering. The thick slurry was washed using deionized water until the filtrate was close to neutral, and then they were dried for 10 h in the oven at 70 °C. White Mg-Al LDH precursors were created, and the precursor particles below 74 μm in size were selected by crushing and screening. The elemental ratios were confirmed by EDS analysis (as shown in Figure S3 and Table S1). The Mg-Al LDO adsorbent powders were produced by the calcination of the precursors in a muffle furnace with an accompanying heating rate of 10 °C min^−1^. When the furnace finally got to the target of 480 °C, the sample was kept inside for 3 h (as shown in Figure S4).

### 2.3. Analysis Methods

The X-ray diffraction (XRD) patterns of samples were collected using Cu Kα radiation at an ambient temperature between 5° and 80° (2θ) with 0.013° step sizes (PANalytical, Malvern, Netherlands, Empyrean). X’Pert High Score Plus was used for analyzing the XRD pattern and calculating the d-value. Fourier-transform infrared spectroscopy (FT-IR) was performed using a Nicolet iS10 FT-IR spectrometer (Thermo Fisher Scientific, Waltham, MA, USA) within 500–4000 cm^−1^. The changes in the surface morphology of the samples were observed using scanning electron microscopy (SEM, Zeiss, Jena, Germany, Sigma 300). A Brunner–Emmett–Teller (BET) analysis (BELSORP Max II, MicrotracBEL, Osaka, Japan) was conducted to investigate the pore structure parameters of the materials before and after the Cl^−^ removal experiments. The samples were pre-treated at 105 °C for 8 h under vacuum and were measured at a temperature of −196.15 °C (77 K). Zeta potential parameters of materials were measured using a Malvern Zetasizer nano series (Malvern Panalytical Ltd., Malvern, UK). Lastly, the thermostability of the materials was measured using a thermogravimetric analyzer (TGA) (Netzsch, STA449 F5, Selb, Germany).

### 2.4. Adsorption Experiments

Batch methods were employed to understand the effect of the Mg/Al molar ratio, calcination temperatures, the calcination time, adsorbent doses, the Cl^−^ initial concentration, the contact time, and the adsorption temperature.

The experiment was launched by transferring 25 mL of the NaCl solution into 100 mL Erlenmeyer flasks using a pipette. A certain amount of the adsorbent was put into the flasks at 293 K. The mixed solution was stirred magnetically at 300–320 r/min for 2 h. The Cl^−^ concentration of the supernatant was detected using an ion chromatograph (Thermo Fisher Scientific). The removal rate calculation formula is as follows [25]:

(1) $$Y=\frac{C_{0}-C_{e}}{C_{0}}\times 100\%\;$$

where *Y* (%) is the Cl^−^ removal rate and *C*_0_ (mg L^−1^) and *C_e_* (mg L^−1^) are the initial and equilibrium concentrations of Cl^−^, respectively.

In the case of pollution, plastic wrap was used during the experiments. The adsorption kinetics were carried out in a specific time, and the adsorption isotherms were plotted at 293 K. All adsorption experiments were repeated, and the average data were used to assess the adsorption performance.

## 3. Results and Discussion

### 3.1. Effect of M^2+^/M^3+^ on the Mg-Al LDO Adsorbent

The structures of the precursor were studied, and the adsorption properties of the Mg-Al LDO adsorbent with different M^2+^/M^3+^ molar ratios were studied. When the molar ratio of M^3+^/ (M^2+^ + M^3+^) was between 0.2 and 0.4 [46], a structured LDH could be acquired.

**Figure 1:**
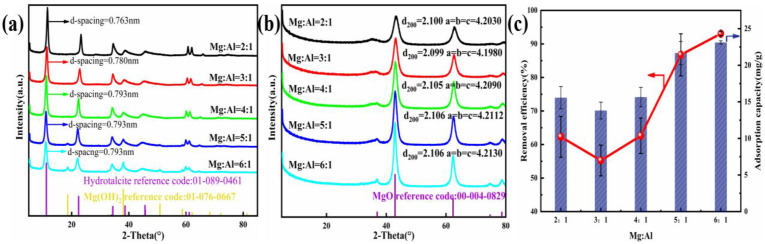
(**a**) XRD pattern of the precursor. (**b**) Diffractograms of the LDO materials. (**c**) Adsorption properties of the adsorbent with different M^2+^/M^3+^.

To research the influence of different Mg/Al molar ratios on Cl^−^ removal, firstly, the composition of the adsorbents was optimized by modifying this ratio. As shown in Figure 1c, Cl^−^ removal efficiency strongly correlates with the rising Mg^2+^ mole ratio of Mg-Al LDO; *n*(Mg^2+^): *n*(Al^3+^) impacts removal efficiency in a significant way. As there was a rise in the molar ratio, the removal efficiency of Cl^−^ firstly decreased but then increased. When *n*(Mg^2+^): *n*(Al^3+^) was 6:1, the Cl^−^ removal was highly competent, at 93.06%. In Figure 1a, the additional crystalline phase that is present in the Mg:Al = 5:1 and Mg:Al = 6:1 sample was allocated to Mg (OH)_2_ [47], whose reflection peaks were at 18.6°, 38.0°, 50.9°, and 58.7°. Accompanying the rise in the ion ratio, the layer spacing of the synthesized materials gradually changed from 0.763 to 0.793 nm and finally appeared to become stable. With the increase in the divalent metal cation content, the charge density of the laminate decreases. The decrease in the stability and the increase in the spacing of the interlayer anions caused by the decrease in the charge density of the laminates are the reasons for the rise in the layer spacing. Combined with the results of material characterization and analysis, the calculation method of layer spacing $\;d_{hkl}=\frac{a}{\sqrt{\frac{4}{3}\left(h^{2}+k^{2}+hk\right)+l^{2}\frac{a^{2}}{c^{2}}}}$ was obtained. The rise in the removal efficiency was imputed to the increase in the relative fraction of the MgO accompanying the gain in the Mg^2+^: Al^3+^ molar ratio. When the LDO was placed into the NaCl solution, the MgO transformed back into the Mg (OH)_2_ phase and adsorbed the other ions to restore the original layered structure [30]. Taking account of the high efficiency and stability of data, the mole ratio of Mg^2+^: Al^3+^ = 6:1 was selected to assess the mechanism of Cl^−^ incorporation by the adsorbent.

XRD patterns and parameters of the LDH precursor are presented in Figure 1a and Table 1. Reflection peaks typically seen in double-layer hydroxide structures were noted in all samples. Reflections peaks at 11.6°, 23.4°, 60.7°, and 62.1° could be indexed to the crystal faces (003), (006), (110), and (113), which are typical to LDH structures [48,49]. In addition, the peaks at 34.9°, 39.4°, and 46.9° corresponded to asymmetric planes at (012), (015), and (018), which reveals the characteristics of hydrotalcite-like compounds [50].

Diffractograms and parameters of LDO are presented in Figure 1b. Reflection peaks typically seen in magnesium oxide were noted in all adsorbents. In addition, accompany the increase in the ion molar ratio, the diffraction peak of magnesium oxide becomes stronger and narrower, which indicates the increase in the metal oxide content. This is also in line with our conclusion that the relative fraction of MgO was responsible for the rise in removal efficiency.

**Figure 2:**
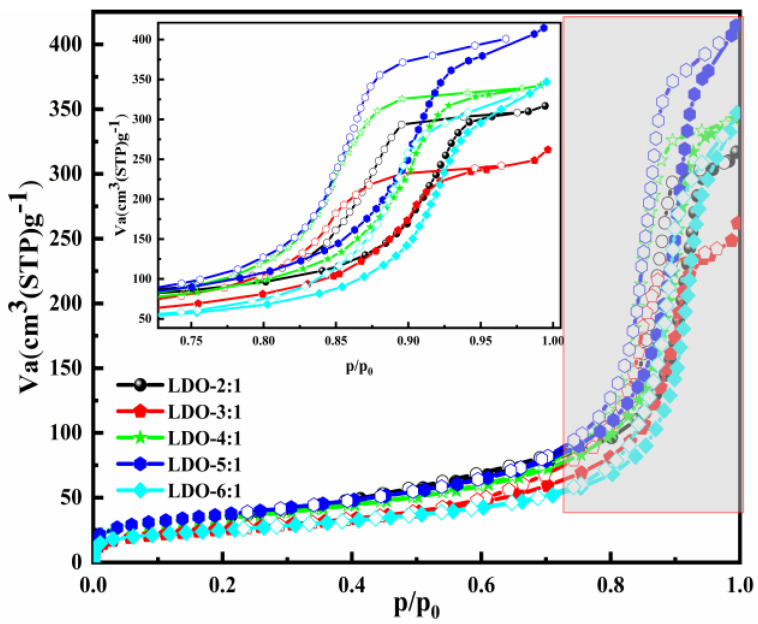
The textural properties of the adsorbents with different ion molar ratios.

The N_2_ adsorption–desorption isotherms and parameters of the LDO materials with different ion molar ratios are presented in Figure 2 and Table 2. Narrow adsorption–desorption hysteresis loops and type Ⅳ isotherm were observed in all samples [51,52]. Accompany the rise in the Mg/Al molar ratio, the average pore diameter increased and reached 23.958 nm when the Mg/Al = 6:1. The larger average pore diameter can expose more active sites and provide favorable conditions for Cl^−^ adsorption.

### 3.2. Adsorption Properties

#### 3.2.1. Effect of the Adsorbent Dose

**Figure 3:**
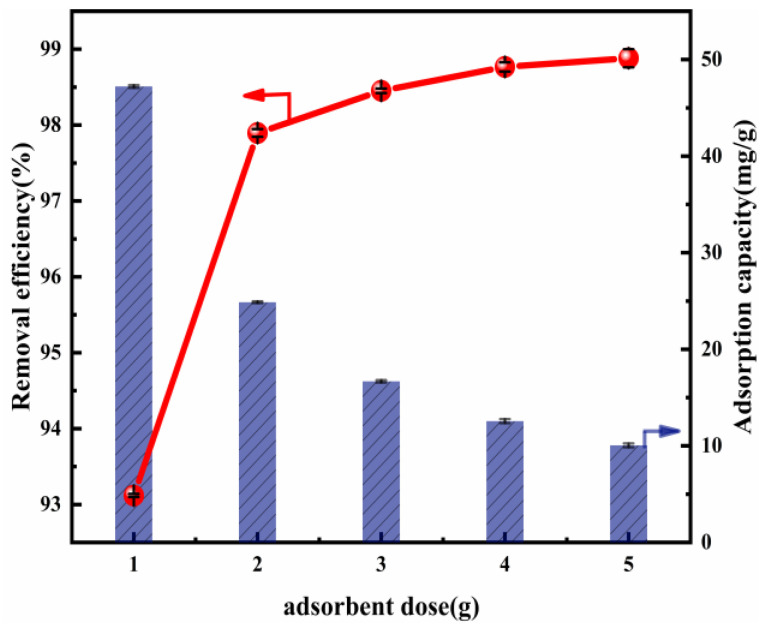
Cl^−^ removal efficiency and adsorption capacity as a function of the adsorbent dosage (ion ratio Mg:Al = 6:1; Cl^−^ initial concentration: 2000 mg L^−1^; adsorption temperature: 293 K; contact time: 13 h).

Figure 3 shows the influence of the variation in the adsorbent dose in Cl^−^ removal. There is enhanced Cl^−^ removal efficiency as the Mg-Al LDO concentration increases. The reason for this may be the increase in accessible active sites for adsorption [50]. When the Mg-Al LDO (Mg:Al = 6:1) dose was 2 g, the removal efficiency of this material reached 97.90% and the adsorption capacity was 24.88 mg/g. This may be linked to the pore diameter, which was obviously larger after calcination (Table S3). The increase in the removal efficiency, which positively shifted from 93.12% to 97.90%, may be explained by reference to the rise in active sites of adsorption due to the Mg-Al LDO dose. That is, with the increase in the material dose, the active sites exposed were sufficient for the adsorption of Cl^−^. Thus, the removal efficiency of Cl^−^ increased during the experiment. Cl^−^ removal efficiency was positively changed by 0.87% (from 97.90% to 98.77%) when there was a more significant increase, of 3 g, in the Mg-Al LDO dose (from 2 to 5 g). This implies that the effect of the dose on the removal of Cl^−^ is insignificant when the amount of adsorbent exceeds 2 g. In addition, accompanying the increase in the material dose from 1 to 5 g, the adsorption capacity negatively changed by 37.16 mg/g (from 47.22 to 10.06 mg/g). When the amount of material increases, the aggregation of the material and competition for Cl^−^ may occur, resulting in a scenario in which the active sites cannot be fully used. Under the condition that the amount of Cl^−^ is consistent, the adsorption capacity decreases. Taking into consideration the Cl^−^ removal efficiency, the impact on adsorption capacity, and economic benefits, we could consider 2 g as the optimal dose to be employed in further research.

#### 3.2.2. Effect of the Initial Cl^−^ Concentration

**Figure 4:**
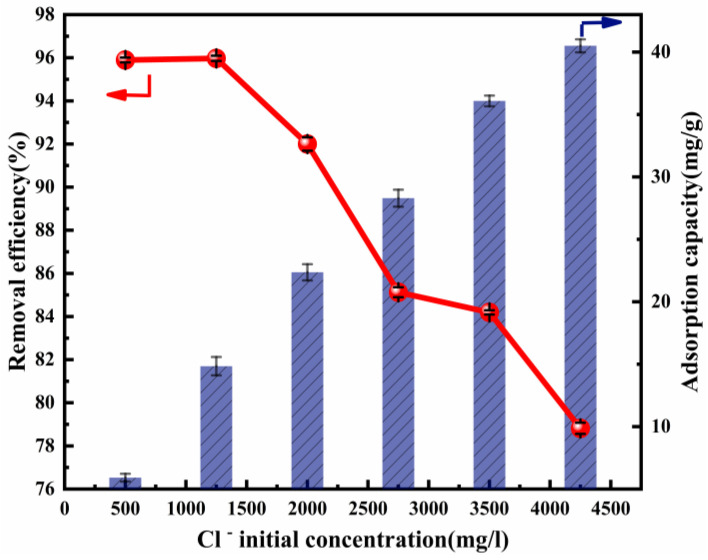
Cl^−^ removal efficiency and adsorption capacity as a function of the Cl^−^ initial concentration (ion ratio Mg:Al = 6:1; adsorbent dose: 2 g; adsorption temperature: 293 K; contact time:13 h).

The initial concentration of Cl^−^ significantly affects its removal efficiency. It is assumed that a high concentration of Cl^−^ is competitive at the adsorption sites [53]. The initial concentration of Cl^−^, which was within the range of 500–4250 mg L^−1^, was used in the adsorption experiment. The influence of the initial concentration of Cl^−^ upon its removal efficiency and adsorption capacity is presented in Figure 4. The adsorption capacity increased rapidly from 5.92 to 40.50 mg/g, accompanying the rise in the initial concentration of Cl^−^. However, the adsorption efficiency then decreased. An explanation for this result may be that there were abundant active sites to be used at a lower Cl^−^ concentration, while there was increased competition between Cl^−^ at a higher Cl^−^ concentration. The experiments above were carried out with a fixed adsorption dose (2 g) and ion ratio (Mg:Al = 6:1). If the amount of adsorbent was increased, more active sites could be provided and the removal efficiency would increase, as can be seen in Figure 3. These results show that Cl^−^ removal could be made much more efficient.

#### 3.2.3. Effect of the Contact Time

**Figure 5:**
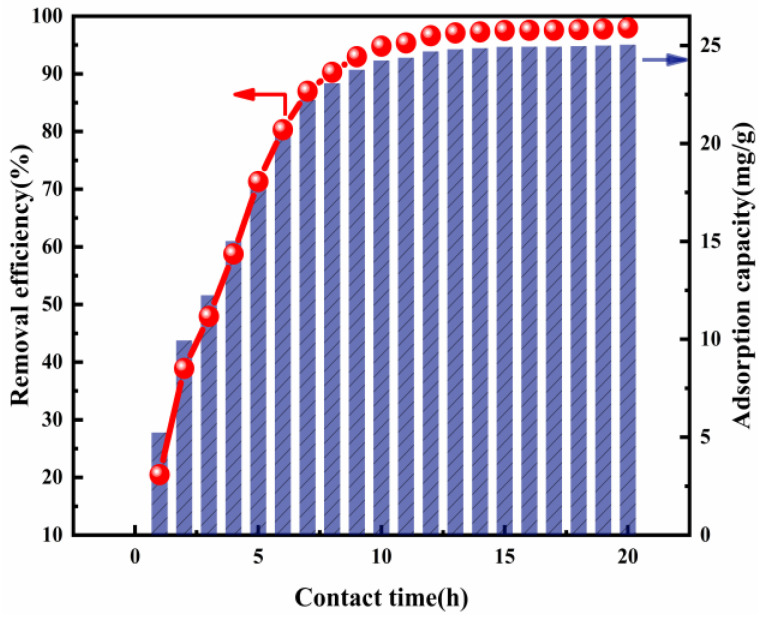
Cl^−^ removal efficiency and adsorption capacity as a function of contact time (ion ratio Mg:Al = 6:1; Cl^−^ initial concentration: 2000 mg L^−1^; adsorbent dose: 2 g; adsorption temperature: 293 K).

Contact time is a key parameter by which to assess the performance of the adsorbent, especially in the progress of actual wastewater treatment, where economic benefits are concerned [25]. The influence when the contact time is raised from 1 to 20 h at 293 K is shown in Figure 5. It can be pointed out that there was a significant impact upon Cl^−^ removal. Cl^−^ removal efficiency increased rapidly from 19.35% to 96.96% as the contact time was increased from 1 to 13 h. This increased especially sharply during the initial 8 h and gradually became almost constant, ranging between about 96.96% and 98.08%, after 13 h. Taking into account the Cl^−^ removal efficiency and economic benefits, the optimal contact time chosen was 13 h, which was also set in the other adsorption experiments.

#### 3.2.4. Effect of the Adsorption Temperature

**Figure 6:**
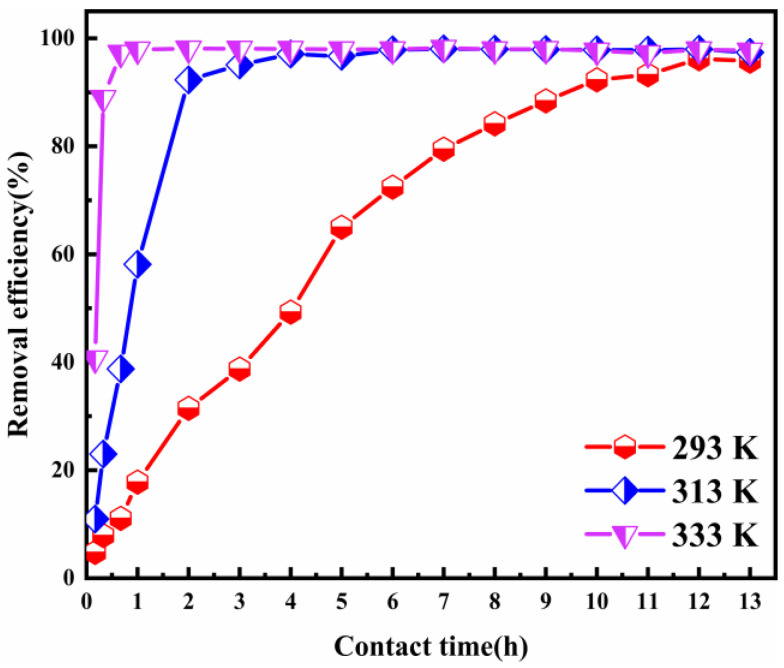
Cl^−^ removal efficiency as a function of the adsorbent dose (ion ratio Mg:Al = 6:1; Cl^−^ initial concentration: 2000 mg L^−1^; adsorbent dosage: 2 g; contact time: 13 h).

The temperature of the solution is an important factor in the adsorption process. Figure 6 shows the impact of the adsorption temperature on Cl^−^ removal. The experimental results were achieved within the temperature range of 293–333 K in the contact time of 13 h and an ion ratio Mg:Al of 6:1. As the adsorption temperature was increased from 293 to 333 K, the Cl^−^ removal efficiency raised sharply. It rose particularly sharply between the contact time of 10 min and 2 h. When the adsorption temperatures were 293, 313, and 333 K, the Cl^−^ removal efficiency was 95.78%, 97.39%, and 97.93%, respectively. The Cl^−^ adsorption equilibrium time was greatly shortened to accompany the rise in the adsorption temperature. The adsorption process reached equilibrium within 1 h, when the adsorption temperature was 333 K. These results indicate that increasing the temperature can speed up the adsorption process.

### 3.3. Adsorption Kinetics

To further understand the relationship between the adsorption capacity and the contact time, the adsorption kinetics were studied at a constant concentration [54]. Three kinetic models, namely the pseudo-first-order kinetic model, the pseudo-second-order kinetic model, and an intraparticle diffusion model, were expressed as follows:

(2) $$\ln\left(q_{e}-q_{t}\right)=\ln q_{e}-k_{1}\times t$$

(3) $$\frac{t}{q_{t}}=\frac{1}{k_{2}q_{e}^{2}}+\frac{t}{q_{e}}$$

(4) $$q_{t}=k_{p}\times t^{\frac{1}{2}}+C$$

where *q_t_* (mg g^−1^) and *q_e_* (mg g^−1^) represent the adsorption amount of Cl^−^ at time t and at equilibrium time, respectively; *t* represents the contact time (min), and *k*_1_ (min^−1^) is the corresponding rate constant of the pseudo-first-order kinetic model. *k*_2_ (g mg^−1^ min^−1^) is the corresponding rate constant of the pseudo-second-order kinetic model. *kp* (mg g^−1^ min^1/2^) is the distribution coefficient responding to the diffusion rate within the particle. *C* is a constant of the particle diffusion [55] corresponding to the boundary layer thickness.

**Figure 7:**
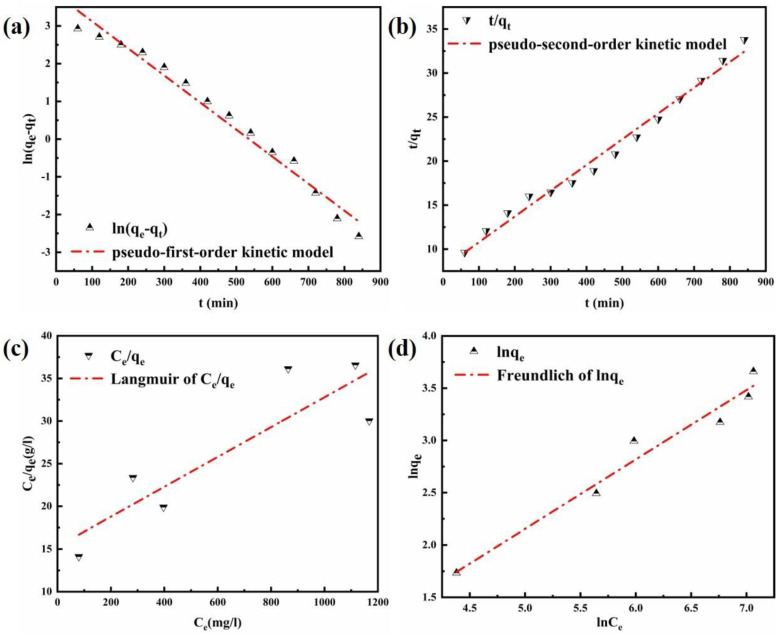
Diagram of (**a**) pseudo−first−order kinetic model and (**b**) pseudo−second−order kinetic model for the adsorption of Cl^−^ on the LDO; (**c**) Langmuir model and (**d**) Freundlich model for the adsorption of Cl^−^ on the LDO.

Figure 7a,b shows the fitting results of the pseudo-first-order kinetic model and the pseudo-second-order kinetic model under an adsorption temperature of 293 K. The calculated parameters are listed in Table 3. As indicated in the results, the pseudo-second-order kinetic model has a higher value of the correlation coefficient (R^2^ = 0.99) compared with the pseudo-first-order kinetic model (R^2^ = 0.98). Furthermore, the theoretical calculated adsorption capacity of the pseudo-second-order kinetic model (*q_e_*, cal) was near to the value of experimental (*q_e_*, exp). That is the pseudo-second-order kinetic model is the most suitable for describing the adsorption of Cl^−^. It was, therefore, suggested that the Cl^−^ adsorption process involves chemical adsorption, which may relate the interactions between Cl^−^ and the laminate of the Mg-Al LDO to this phenomenon. 

**Figure 8:**
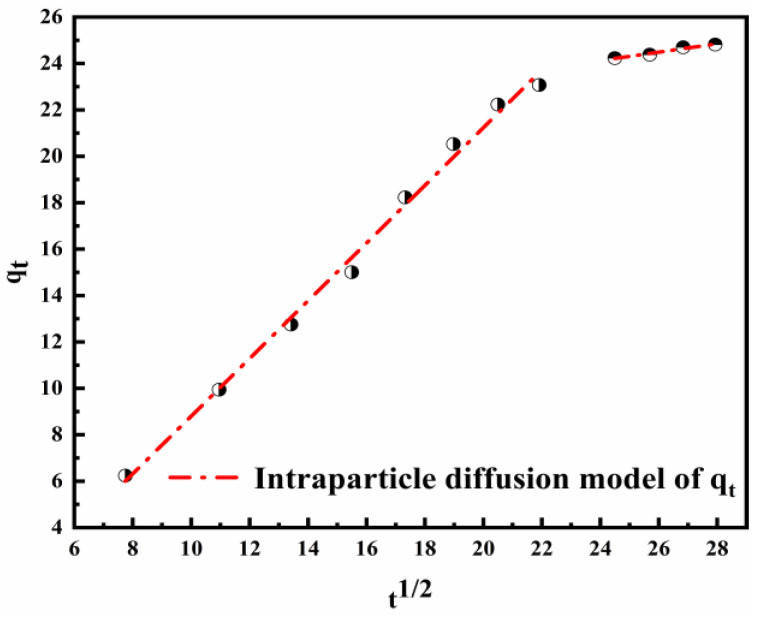
Diagram of the intraparticle diffusion model for the adsorption of Cl^−^ on the LDO.

The adsorption results were used to simulate the intraparticle diffusion model. It is a diffusion phenomenon that controls the adsorption process when the fitting plot is linear. Figure 8 shows the plot of qt vs. t^1/2^, and the parameters of the intraparticle diffusion model calculated are represented in Table 4. The adsorption of Cl^−^ is a complicated process because the linear plot does not pass through the origin. The first stage concerns the adsorption and diffusion of Cl^−^ on the surface of the material. The diffusion rate is faster than expected. The second stage is related to the process of intraparticle diffusion. Taking into consideration the rate constants, it can be concluded that the secondary step was the rate controlling step in the process of adsorption. This may be due to the reduction in effective active sites and the resulting thicker boundary layer [56].

### 3.4. Adsorption Isotherm

Simulating the experimental data using the Langmuir isotherm model and the Freundlich isotherm model can help us to further understand the mechanism of Cl^−^ removal. The Langmuir isotherm assumes that, based on the monolayer adsorption, the adsorption of adsorbate molecules on the active site is uniform, while the Freundlich isotherm assumes the adsorption is multilayer and heterogeneous; the model is applicable to chemical adsorption [25,55]. The expressions of these models can be respectively presented as follows:

(5) $$\frac{C_{e}}{q_{e}}=\frac{1}{q_{m}K_{L}}+\frac{C_{e}}{q_{m}}$$

(6) $$\ln q_{e}=\ln K_{F}+\frac{1}{n}\times \ln C_{e}$$

where *q_e_* (mg g^−1^) and *q_m_* (m mol g^−1^) represent the equilibrium capacity and the maximum adsorption capacity, respectively. *C_e_* (mg L^−1^) represents the equilibrium of the Cl^−^ concentration. The equilibrium adsorption constant *KL* (L mg^−1^) is used to express the anions’ affinity to the binding sites, the Freundlich constant *KF* is associated with the adsorption capacity, and *n* is named as the heterogeneity factor.

Comparing the Langmuir fitting results with the Freundlich isotherm models (as shown in Figure 7c,d and Table 5), it can be observed that the adsorption of Cl^−^ onto the material better coincides with the Freundlich model (R^2^ = 0.96), indicating that there was a co-adsorption process [55]. Where the value of *n* is greater than 1, it can be concluded that sorption is favorable. After a combined analysis of the adsorption kinetics, it can be concluded that the adsorbent is appropriate when used in practical applications.

The thermodynamic data can be calculated using the following equations:

(7) $$K=\frac{C_{0}-C_{e}}{C_{e}}$$

(8) $$\ln K=\frac{\Delta S^{0}}{R}-\frac{\Delta H^{0}}{RT}$$

(9) $$\Delta G^{0}=-RT\ln K$$

*K* is the equilibrium constant of the process, and *R* (J mol^−1^ K^−1^), with a value of 8.314, is the universal gas constant. Δ*G*^0^ (kJ mol^−1^) represents the change in free energy, Δ*S*^0^ (J mol K^−1^) is entropy change, and Δ*H*^0^ is enthalpy change.

Results are displayed in Table S5. The value of Δ*G*^0^ was negative, and it suggests that the removal process was spontaneous. The positive Δ*H*^0^ indicates the endothermic parts of this process, and the negative value of Δ*S*^0^ confirms that the degree of chaos had increased. In the process of chloride ion removal, Cl^−^ was absorbed to reconstruct the bimetallic hydroxide layered structure and the action of ion exchange further enhanced the process.

### 3.5. Recyclability of LDO Adsorbents

**Figure 9:**
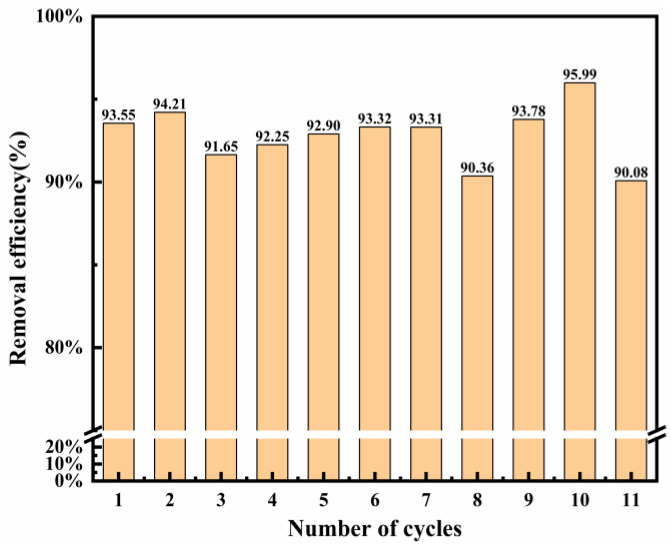
Reusability of LDO (calcinated at 480 °C) on Cl^−^ adsorption (Cl^−^ initial concentration: 2000 mg L^−1^; adsorbent dosage: 2 g; adsorption temperature: 293 K; contact time: 13 h).

The reusability of the adsorbent is an important indicator of whether the adsorbent is economical. Thus, its reusability was assessed by conducting repetitive experiments within the same conditions. Figure 9 shows the recycling time on Cl^−^ removal in the wastewater. Mg-Al LDO was recovered in the layered structure in the aqueous solution, which provides more active sites for the adsorption of Cl^−^. In this work, Mg-Al LDO Cl^−^ was calcinated at 480 °C to obtain the adsorbent and then the re-calcinated adsorbent was used for Cl^−^ adsorption again, within the same conditions. The Mg-Al LDO still exhibited good adsorption performance (more than 90%) for Cl^−^ after 11 adsorption–desorption cycles. This indicates that the synthesized LDO has a good recycling performance and can reduce the costs of the process to a certain extent. Compared to other materials, the Mg-Al LDO has the highest Cl^−^ removal efficiency (Table 6). The restoration of the layered structure and the combined action of ion exchange allow for better removal of Cl^−^. The different synthesis method and the experimental environment may result in different properties of materials. Therefore, the Mg-Al LDO studied in this work exhibits better removal efficiency of Cl^−^.

### 3.6. Mechanism for Cl^−^ Removal

**Figure 10:**
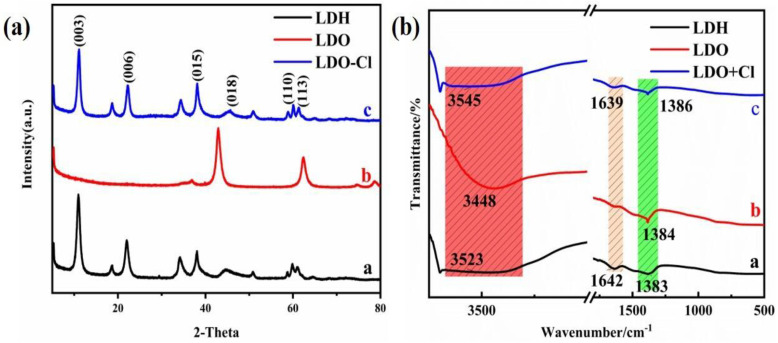
(**a**) X-ray diffraction analysis; (**b**) FT-IR spectra of the samples.

**Figure 11:**
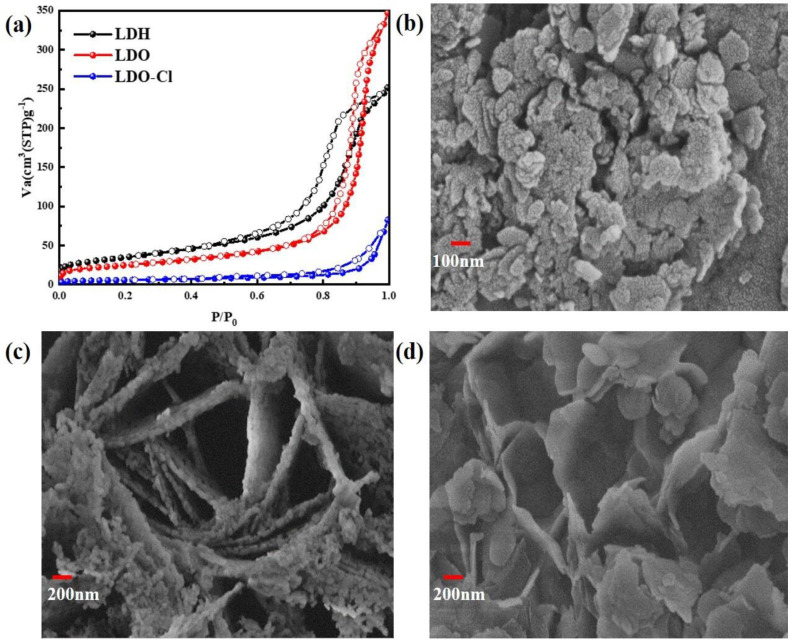
(**a**) N_2_ adsorption—desorption isotherms; (**b**–**d**) SEM images of the samples.

XRD patterns of the Mg-Al LDH precursor, Mg-Al LDO, and Mg-Al LDO Cl^−^ are demonstrated in Figure 10a. The XRD pattern of the Mg-Al LDO Cl^−^ is akin to that of the precursor, presenting the typical peaks of hydrotalcite. However, (003), (006), (015), and other characteristic peaks corresponding to Mg-Al LDO have disappeared, showing the characteristic peak of the metal oxide [47]. The characteristic peaks of hydrotalcite have reappeared in Mg-Al LDO Cl^−^, which is consistent with the reconstruction of the layered structure [59]. It is observed that the reflection of (003) has shifted to a higher 2θ angle and that the d-spacing has decreased from 0.793 nm for Mg-Al LDH to 0.786 nm for Mg-Al LDO Cl^−^ (as shown in Table S2). The smaller size of the intercalated anion of Mg-Al LDO Cl^−^ is clearly seen when compared to the Mg-Al LDH precursor [31,60]. The thickness of the laminate was 0.48 nm [61], and the gallery height of the samples can be achieved by subtracting the thickness of the metal hydroxide laminate from the interlayer spacing, which is approximately 0.313 nm for the Mg-Al LDH precursor, while for Mg-Al LDO Cl^−^, it is 0.306 nm and is larger than the ion radius of chloride (0.167 nm). It can be concluded that the space resistance of chloride is small during the process of entering the laminate to reconstruct the layer structure. At the same time, it provides the right conditions for multi-layer adsorption of Cl^−^. From the results of N_2_ adsorption–desorption, sufficient pore volume and larger average pore diameter can be observed and expose more active sites, which provide favorable conditions for Cl^−^ adsorption. The diagram of the SEM spectrum can also confirm this conclusion (as shown in Figure 11c). The lattice parameters of the samples a and c are given in Table S2.

The FT-IR spectra of the samples are displayed in Figure 10b. In all the samples, broad intense bands observed around 3448–3545 cm^−1^ are associated with the stretching vibration of the hydroxyl groups [45,62], and the characteristic absorption band around 1385 cm^−1^ is scribed to the antisymmetric stretching mode of NO_3_^−^ in the interlayer [30]. Characteristic peaks shown at 1640 cm^−1^ are attributed to the bending vibration of the interlayer molecular water [63], which was lost after the calcination process. This confirms the decomposition of the interlayer water. In addition, the slight decrease in 1385 cm^−1^ implies that there is a partial decomposition of the interlayer anions. After the adsorption of Cl^−^, the band at 1383 cm^−1^ shifts to higher frequencies (to 1386 cm^−1^). This may be explained by the influence of Cl^−^ on its reconstructed structure. The bending vibration of molecular water reappears at 1639 cm^−1^, and the schematic diagram of the FT-IR spectrum is similar to that of the precursor, which demonstrates that the layer structure has been well reconstructed.

Figure 11a shows the N_2_ adsorption–desorption isotherms of materials. Nitrogen adsorption–desorption isotherms were used to examine the surface area and porosity of the Mg-Al LDH precursor, the Mg-Al LDO adsorbent, and Mg-Al LDO Cl^−^ samples. As shown in Table S3, the total pore volume and the average pore diameter of Mg-Al LDO were 0.5261 cm^3^ g^−1^ and 23.958 nm, while the measurements for Mg-Al LDH were 0.3843 cm^3^ g^−1^ and 12.269 nm. An obvious increase in the total pore volume and porosity of Mg-Al LDO was observed, which provides a favorable condition for Cl^−^ adsorption. The specific surface area of Mg-Al LDH (87.84 m^2^ g^−1^) increased to 125.27 m^2^ g^−1^ after calcination and decreased again to 20.253 m^2^ g^−1^ after Cl^−^ adsorption. Similar trends were seen in the total pore volume and the average pore diameter after the adsorption of Cl^−^ on Mg-Al LDO (as shown in Table S2). The significant decrease in the specific surface area of Mg-Al LDO Cl^−^ when compared to Mg-Al LDH is borne by different anion species in the interlayer and by the transformation of the crystal structure.

According to the IUPAC classification [51], a type IV isotherm and an H2 hysteresis loop can be observed of the precursor (as shown in Figure 11a). The presence of mesopores and capillary condensation is the cause of this phenomenon [64,65]. For Mg-Al LDO, a narrow adsorption–desorption hysteresis loop was observed when the relative pressure was above 0.7. This hysteresis loop was determined to be a type H1 hysteresis loop with typical tubular capillary pores. After the adsorption of Cl^−^, the Mg-Al LDO Cl^−^ material exhibits type III adsorption isotherms and type H3 adsorption loops [66]. This indicates that the pore structure of the Mg-Al LDO material after the adsorption of Cl^−^ includes cleavage-type pores, which are formed by the accumulation of mutually inclined sheets. The description of this phenomenon is consistent with the results of the scanning electron microscope test. The structure of the material changes after the adsorption of chloride ions, indicating that the reconstruction of the layer structure plays an important role in the removal of Cl^−^.

Figure 11b shows the SEM measurement of the Mg-Al LDH precursor. Figure 11c shows the SEM measurement of the Mg-Al LDO, and Figure 11d shows the SEM measurement of the Mg-Al LDO Cl^−^. These clearly show that the structure of the precursor is stacked in layers. The pores between the Mg-Al LDO layers were detected after calcination, which provides favorable conditions for the removal of Cl^−^ and is consistent with the calculation result of BET. The irregular and flaky and loose stacking structure of Mg-Al LDO Cl^−^ was observed after the adsorption of Cl^−^.

Figure S5 shows the thermal gravimetric analysis of Mg-Al LDH. Three stages of weight loss were observed. The first weight loss stage (14.62%) occurred between room temperature and 245 °C, where adsorbed water on the crystallite surface and crystallized water between the cationic layers were removed. The second weight loss stage (18.17%), which occurred between 245 and 343 °C, is associated with the thermal decomposition of the hydroxyl (OH^−^) groups and accompanied the decomposition of part of nitrate (NO_3_^−^) species. Finally, the last stage of weight loss (15.51%) occurred from 343 °C to 700 °C and corresponds to the loss of nitrate species [45].

The zeta potential has a significant impact on a suspension’s stability. This is because it can provide precise information about the reason for dispersion or aggregation, which has become one of the most important factors influencing the stability of the suspension [50]. The zeta potential of the Mg-Al LDO is positively charged at 6.8 mv (as shown in Table S4). Due to the low surface electrical properties, Cl^−^ can be accelerated into entering the interlayer. After stabilization, the zeta potential of the Mg-Al LDO Cl^−^ increases to 37.12 mv, indicating that the Mg-Al LDO Cl^−^ is much more stable.

The comparative adsorption capacity and anion exchange capacity is shown in Table S6. It can be clearly observed that the adsorption capacity is larger than the anion exchange capacity. That is, the synergistic effect of the reconstruction of the layered structure and the ion exchange enables a more efficient removal of Cl^−^. It is the adsorption of negative anions to reconstruct the layer structure that dominates the process.

**Figure 12:**
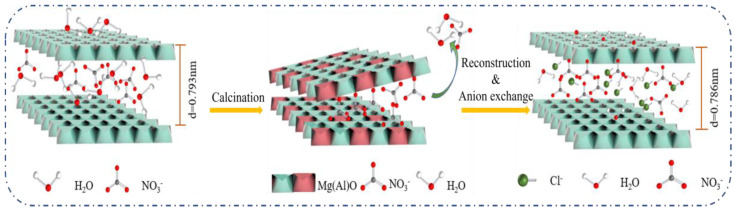
Schematic diagram of the removal mechanism of Mg-Al LDO.

According to the experimental results of adsorption properties and systematically characterization, the process of Cl^−^ removal can be described as follows (as illustrated in Figure 12): the positively charged surface of Mg-Al LDO enhances the electrostatic attraction between the adsorbent and the negatively charged Cl^−^. Sufficient pore volume and larger average pore diameter expose more active sites, which provide favorable conditions for Cl^−^ adsorption. During the reconstruction process, it is deduced that some Cl^−^ adsorbed on the edge rebuild the layer structure first. With the enhanced electrostatic attraction provided by the high charge density laminate, other Cl^−^ enter the layer and are exchanged with the remaining NO_3_^−^, resulting in the efficient removal of Cl^−^.

## 4. Conclusions

In this paper, a Mg-Al LDH precursor was successfully synthesized using an improved coprecipitation method, which considered the effect of M^2+^/M^3+^. The Mg-Al LDO adsorbent was obtained by the calcination of the precursor. The characterization results showed that the adsorbent had a rich pore structure, that Cl^−^ successfully entered the interlayer bonding the laminate to reconstruct the layered structure, and exchange with nitrate remained in the interlayer. The adsorption experimental results suggest that the Mg-Al LDO material exhibits a significant adsorption capacity for Cl^−^. The experiments found that the optimal contact time is 13 h. The optimized dosage of adsorbent is 80 g L^−1^, and the value of the adsorption capacity is up to 24.82 mg g^−1^. Additionally, the Cl^−^ adsorption on to the Mg-Al LDO fits well with the pseudo-second-order model and the Freundlich model. Overall, the adsorbent exhibited a better regeneration performance too. In conclusion, Mg-Al LDO is a promising material for Cl^−^ elimination.

## Figures and Tables

**Table 1:** Layer spacing of the crystal plane under different ratios of materials.

	Mg:Al = 2:1	Mg:Al = 3:1	Mg:Al = 4:1	Mg:Al = 5:1	Mg:Al = 6:1
d-spacing/nm	0.763	0.780	0.793	0.793	0.793
2θ/°	11.593	11.335	11.152	11.152	11.152
a	0.307	0.305	0.308	0.308	0.308
c	2.295	2.340	2.378	2.378	2.378

**Table 2:** Specific surface area, total pore volume, and average pore diameter of the LDO with different ion molar ratios.

Materials	Specific Surface Area (m^2^ g^−1^)	Total Pore Volume (cm^3^ g^−1^)	Average Pore Diameter (nm)
LDO–2:1	128.311	0.49	15.153
LDO–3:1	91.889	0.39	17.015
LDO–4:1	113.943	0.53	17.494
LDO–5:1	130.425	0.63	19.471
LDO–6:1	121.074	0.52	24.081

**Table 3:** Pseudo-first-order and pseudo-second-order kinetic model parameters.

qe, exp	Pseudo−First−Order Kinetic Model			Pseudo−Second−Order Kinetic Model		
mg g^−1^	k_1_(min^−1^)	qe (mg g^−1^)	R_1_^2^	k_2_ (g mg^−1^ min^−1^)	qe (mg g^−1^)	R_2_^2^
24.94	0.01651	46.36	0.98	0.00011	34.14	0.99

**Table 4:** Parameters of the intraparticle diffusion model.

Intraparticle Diffusion Model					
kp_1_ (mg g^−1^·min^1/2^)	C_1_	R^2^	kp_2_ (mg g^−1^·min^1/2^)	C_2_	R^2^
1.2434	−3.6289	0.99	0.1802	19.8046	0.97

**Table 5:** Langmuir and Freundlich isotherm model parameters.

Langmuir Isotherm Model			Freundlich Isotherm Model		
Q_0_ (mg g^−1^)	KL (L mg^−1^)	R^2^	KF (mg g^−1^) (L mg^−1^)^1/n^	*n*	R^2^
57.14	0.0011	0.78	0.312	1.51	0.96

**Table 6:** Comparative study of the removal efficiency of LDO against other reported sorbents for Cl^−^.

Sorbent	Removal Efficiency	Reference
Mg_0.80_Al_0.20_O_1.1_	14.95%	[42]
Double hydrous oxide (Fe_2_O_3_·Al_2_O_3_·xH_2_O)	23.93%	[57]
Zn-Al oxide (Zn/Al = 3.0)	32%	[44]
Mg-Al oxide (Mg/Al = 3.0)	75.41%	[58]
Mg-Al oxide (Mg/Al = 3.0)	96%	[44]
Mg-Al LDO (Mg/Al = 6.0)	97.09%	This work

## Data Availability

All the data is available in this manuscript.

## Supplementary Materials

The following supporting information can be downloaded at: https://www.mdpi.com/article/10.3390/nano12050846/s1, Figure S1: Cl^−^ removal efficiency and adsorption capacity, as a function of the calcination temperature (adsorbent dosage: 2 g; Cl^−^ initial concentration: 2000 mg L^−1^, adsorption temperature: 293 K, contact time: 13 h); Figure S2: Cl^−^ removal efficiency and adsorption capacity, as a function of the calcination time (calcination temperature: 480℃, adsorbent dosage: 2 g; Cl^−^ initial concentration: 2000 mg L^−1^, adsorption temperature: 293 K, contact time: 13 h); Figure S3: EDS images of (a) LDH−2: 1; (b) LDH−3: 1; (c) LDH−4: 1; (d) LDH−5: 1; (e) LDH−6: 1; Figure S4: Preparation process of Mg-Al-LDO; Figure S5: TGA curve of Mg-Al-LDH; pristine LDH; Figure S6: Standard curve of Cl^−^ determined by Ion Chromatograph; Table S1: Concentration ratio of Mg/Al in different samples; Table S2: XRD Parameters of Mg-Al-LDH, Mg-Al-LDO and Mg-Al-LDO-Cl^−^; Table S3: Specific surface area, total pore volume, and average pore diameter of Mg-Al-LDH, Mg-Al-LDO, Mg-Al-LDO-Cl^−^; Table S4: Zeta potential of Mg-Al-LDH, Mg-Al-LDO, Mg-Al-LDO-Cl^−^; Table S5: Thermodynamic data of chloride adsorption; Table S6: Comparative of adsorption capacity and anion−exchange capacity on Cl^−^ removal.
