# Supplementary material for: The Performance and Mechanism of a Mg-Al Double-Layer Oxide in Chloride ion Removal from an Aqueous Solution
Source: Nanomaterials (Basel). 2022 Mar 2;12(5):846. doi: 10.3390/nano12050846 (PMC8912365; doi:10.3390/nano12050846)
Supplement: Supplementary file 1 [file nanomaterials-12-00846-s001.zip › nanomaterials-1577165-supplementary.pdf]

Supplementary Materials

The Performance and Mechanism of a Mg-Al Double-Layer Oxide in Chloride ion Removal from an Aqueous Solution

Xueqin Xu [1], Peng Li [2], Shichong Yang [2], Tong Zhang [2], Xiangke Han [2], Guoli Zhou [2,*], Yijun Cao [2,*] and Daoguang Teng [2]

[1] Henan Province Industrial Technology Research Institution of Resources and Materials, Zhengzhou University, Zhengzhou 450001, China; xxq18838180617@163.com

[2] School of Chemical Engineering, Zhengzhou University, Zhengzhou 450001, China; zdhglipeng@zzu.edu.cn (P.L.); yscofzzu@163.com (S.Y.); yoygat@163.com (T.Z.); xiangkehan@163.com (X.H.); teng_daoguang@zzu.edu.cn (D.T.)

* Correspondence: zglcumt@126.com (G.Z.); yijuncao@126.com (Y.C.); Tel.: +86-371-6778-1081 (G.Z.); +86-371-6773-9808 (Y.C.)

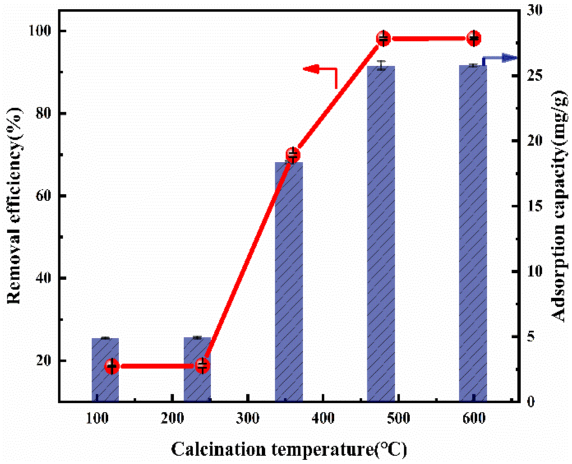

Figure S1. Cl^- removal efficiency and adsorption capacity, as a function of the calcination temperature (adsorbent dosage: 2 g; Cl^- initial concentration: 2000 mg L^{-1}, adsorption temperature: 293 K, contact time: 13 h).

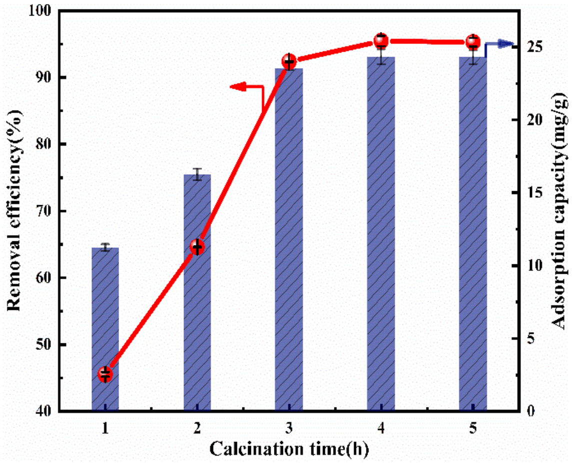

Figure S2. Cl^- removal efficiency and adsorption capacity, as a function of the calcination time (calcination temperature: 480℃, adsorbent dosage: 2 g; Cl^- initial concentration: 2000 mg L^{-1}, adsorption temperature: 293 K, contact time: 13 h).

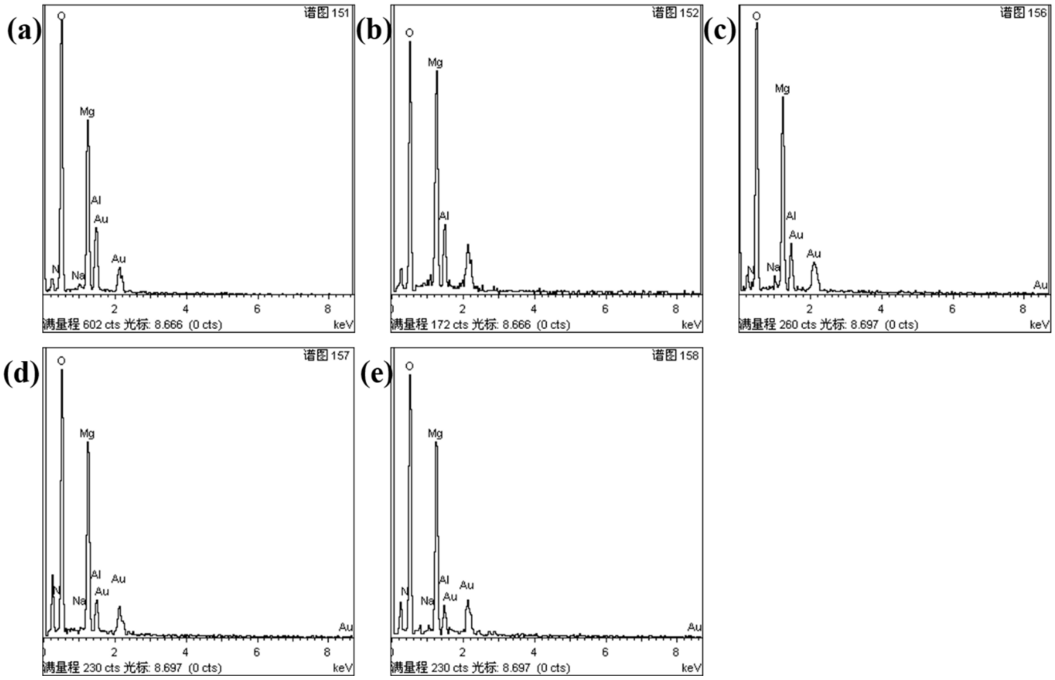

Figure S3. EDS images of (a) LDH–2: 1; (b) LDH–3: 1; (c) LDH–4: 1; (d) LDH–5: 1; (e) LDH–6: 1.

Table S1. Concentration ratio of Mg/Al in different samples.

Materials	Concentration ratio of Mg/Al
LDH–2:1	2.59
LDH–3:1	3.36
LDH–4:1	4.11
LDH–5:1	5.57
LDH–6:1	6.85

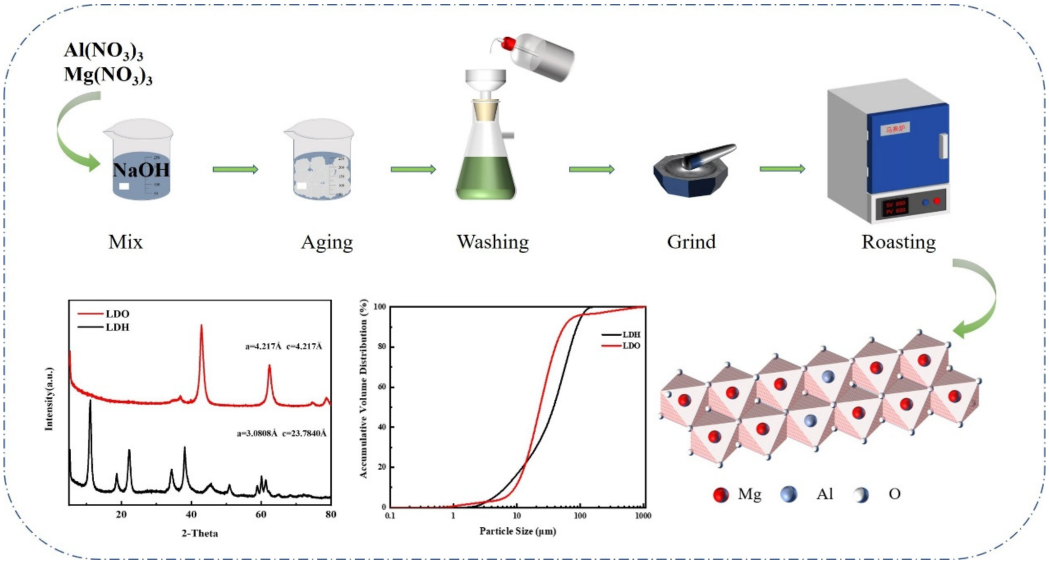

Figure S4. Preparation process of Mg-Al-LDO.

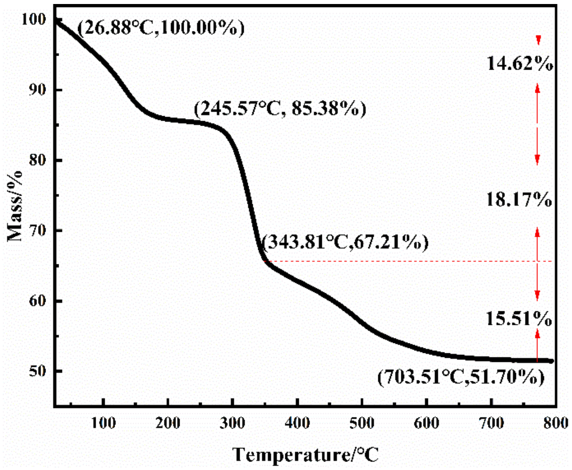

Figure S5. TGA curve of Mg-Al-LDH; pristine LDH.

Table S2. XRD Parameters of Mg-Al-LDH, Mg-Al-LDO and Mg-Al-LDO-Cl^-.

Mg-Al-LDH		Mg-Al-LDO		Mg-Al-LDO-Cl^-	
d–Spacing/nm	0.793	d_{111}/nm	0.244	d–Spacing/nm	0.786
2θ/°	11.152	d_{200}/nm	0.211	2θ/°	11.248
a	0.308	d_{220}/nm	0.149	a	0.307
c	2.378	a=b=c	0.422	c	1.550

Table S3. Specific surface area, total pore volume, and average pore diameter of Mg-Al-LDH, Mg-Al-LDO, Mg-Al-LDO-Cl^-.

materials	Specific surface area ($m^2\ g^{-1}$)	Total pore volume ($cm^3\ g^{-1}$)	Average pore diameter (nm)	r_{Kelvin} (nm)
Mg-Al-LDH	87.841	0.38	12.269	
Mg-Al-LDO	125.273	0.53	23.958	0.621
Mg-Al-LDO-Cl^-	20.253	0.12	23.096	

Notes: the calculated r_{Kelvin} of the three samples are 0.621 nm, respectively.

Table S4. Zeta potential of Mg-Al-LDH, Mg-Al-LDO, Mg-Al-LDO-Cl^-.

materials	Mg-Al-LDH	Mg-Al-LDO	Mg-Al-LDO-Cl^-
Zeta(mv)	21.22	6.80	37.12

Table S5. Thermodynamic data of chloride adsorption.

Temperature (K)	ΔG^0 ($kJ \cdot mol^{-1}$)	ΔH^0 ($kJ \cdot mol^{-1}$)	ΔS^0 ($J \cdot mol \cdot K^{-1}$)	E_a ($kJ \cdot mol^{-1}$)
293	–7.60			
313	–9.42	13.14	71.20	13.14
333	–10.42			

Table S6. Comparative of adsorption capacity and anion–exchange capacity on Cl^- removal.

Samples	Adsorption capacity (mg/g)	Anion–exchange capacity (mg/g)
Mg-Al-2-LDOs	16.30	0.21
Mg-Al-3-LDOs	12.80	0.27
Mg-Al-4-LDOs	16.59	0.21
Mg-Al-5-LDOs	21.68	0.15

Mg-Al-6-LDOs	23.12	0.15

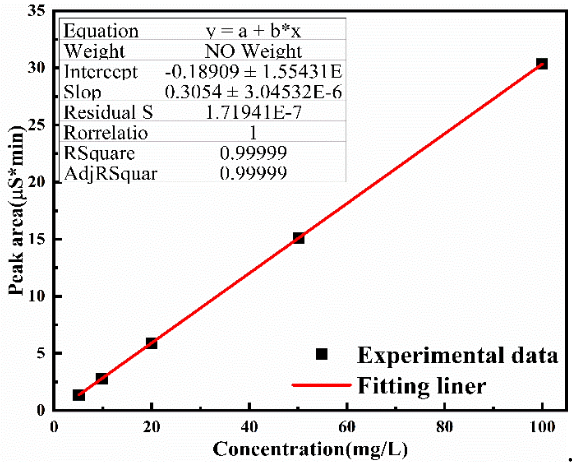

Figure S6. Standard curve of Cl^- determined by Ion Chromatograph.
